# Barriers and facilitators to primary care staff conducting research – a qualitative systematic review

**DOI:** 10.1080/13814788.2025.2539777

**Published:** 2025-08-13

**Authors:** Zoe Edwards, Michael Tatterton

**Affiliations:** a School of Pharmacy & Medical Sciences, University of Bradford, Bradford, West Yorkshire, UK; b Affinity Care Primary Care Network, Bradford, West Yorkshire, UK; c School of Nursing & Healthcare Leadership, University of Bradford, West Yorkshire, UK

**Keywords:** Research, primary care, staff, barrier, facilitator

## Abstract

**Background:**

Research is vital for progress and development of healthcare and may help relieve current health service pressures through improvements and efficiencies. Research in primary care is not well established and is not part of routine practice. This study aims to investigate the barriers and facilitators to primary care staff conducting research.

**Method:**

A systematic literature review was conducted in CINAHL, Medline, APA, PsycInfo, AHMED and EMBASE from inception to April 2023. Searches were for studies involving clinical or non-clinical staff working in primary care where barriers or facilitators to conducting research were examined.

**Results:**

Twenty-one studies were included from 2000 to 2022. The QuADs quality appraisal method found that papers were of varying, often low quality. Five themes were found – research beliefs & understanding, time, funding & recognition, skills & knowledge, administration & support, ethics & understanding and communication & people. Staff thought research useful but optional and were impeded by time and funding. They need training and support to carry out research. Communication from the researchers before, during and after study completion would prevent problems and lead to more research participation in the future.

**Conclusion:**

Improved communication at all stages would serve as a facilitator to primary care staff conducting research. Clear, appropriate training for all staff would allow them to complete appropriate tasks for their roles and prevent one individual taking full responsibility. Embedding research in primary care with protected time and resources to complete it would remove barriers to taking part.

## KEY MESSAGES

Barriers and facilitators to staff conducting primary care research include time, funding and support.

Clear communication between researchers and staff before, during and after studies would facilitate involvement.

Research training would allow appropriate staff members to do appropriate tasks therefore embedding research into the whole primary care team.

## Introduction

Healthcare depends upon research, and improvements in healthcare cannot be achieved without research [1]. Whether developments come in the form of new medicines, new pathways, prevention or earlier diagnosis, all these depend upon research [2]. It is well documented that healthcare organisations which take part in research provide improved care to their patients with better outcomes and a higher level of patient safety [3–5].

Research is routinely carried out in secondary care and is accepted as being part of most healthcare professional roles [6]. In primary care, research is less well-established, and in 1999 an Australian study found that publication rates for primary care research were about a hundred-times less than those in secondary care [7]. Although improvements have been made in the past 20 years, research is still not expected of primary care staff. General practices (GPs) are encouraged to take part in research, but it is very much optional and not mandatory. Hospitals and NHS Integrated Care Boards (ICBs) have departments dedicated to research delivery, but the smaller groups of GP practices or individual practices often do not have this and rely on local adopters and good practice to carry out research [8]. Often research does not happen in communities that have the greatest healthcare need, or the potential to benefit the most [9].

Research in primary care provides vital information about whether treatments and interventions are effective and worthwhile [2]. Primary care is, as its name states, the first port of call for most patients accessing healthcare and has four times the number of patient contacts as secondary care [1]. In a post-COVID landscape the pressures on healthcare are huge with staffing problems and strikes adding to an already overstretched service. A recent poll showed that most UK GPs currently deliver more than the recommended safe amount of patient contacts per week leaving little room in their working week for extra activity [10].

Primary care research often consists of conducting searches for eligible patients, inviting them to take part and then sometimes carrying out an intervention with them or altering their care pathway depending on the study. Some primary care settings also carry out drug trials.

Much literature is available on the difficulties of recruiting patients and staff to take part in research but this paper is not concerned with this. This study focuses on the barriers and/or facilitators to primary care staff conducting research. There are several studies looking at these barriers and/or facilitators but a systematic review has not been carried out in this area.

### Aims and objectives

The aim is to investigate the barriers and facilitators to primary care staff conducting research by carrying out a systematic review.

The review question is ‘What are the barriers and facilitators to primary care staff conducting research?’ and was developed using the Population, Intervention, Comparison and Outcomes (PICO) criteria to ensure all elements of the question were considered [11]. No protocol has been written or submitted for this review and it has not been registered.

## Methods

The databases CINAHL, Medline, APA, PyscInfo, AHMED and EMBASE were searched in between February and April 2023 by ZE. Searches were for records from database inception until the present day to ensure that data collection includes all studies which have been carried out on the subject.

The PICO criteria were expanded to capture all alternative words and Medical Subject Headings (MeSH). The Boolean search found in Appendix 1 was then employed in each database. Reference lists from resulting papers were also screened for suitability.

### Eligibility criteria

The following criteria were applied to the studies identified in the literature search:

Inclusion criteria:

- Studies were solely or partially based in primary care.
- Exploring clinical or non-clinical staff involvement in research were part of the aims.
- Barriers and/or facilitators to voluntary involvement in research.

Exclusion criteria:

- Settings other than primary care.
- Patient participation in research was the sole focus of the study.
- Not research (opinion pieces/reviews of existing research/educational).
- Studies solely exploring student research activity.

Only studies with the full texts available in the English language were included although an English translation was acceptable. Studies were reviewed by both ZE and MT.

### Quality assessment

The quality of included studies was assessed using Quality Assessment with Diverse Studies (QuADS) which is a tool for reporting quality in systematic reviews of mixed or multi-method studies [12]. This tool was selected due to its reliability for health-related research and the range of different methods used by included studies. Assessing quality of mixed methods studies allows understanding of bias and enables evaluation of validity of study findings [12].

### Data extraction and reporting

Data from the identified papers were extracted into an Excel spreadsheet by ZE in a standardised form. This included methods, participants, intervention, setting, barriers and facilitators. Data were recorded on barriers and facilitators for each study along with numbers of participants, participant groups, settings and interventions to aid analysis.

### Data analysis

Thematic analysis was then used to identify themes in the data. This method was used as it is structured, flexible, efficient and allows for both differences and similarities of data to be highlighted [13]. The five phases of thematic analysis were employed. Initially ZE and MT familiarised themselves with the data, initial coding was then carried out before searching for themes. The themes were reviewed and then names were generated for the themes before producing the written report [13].

## Results

**Figure 1.**
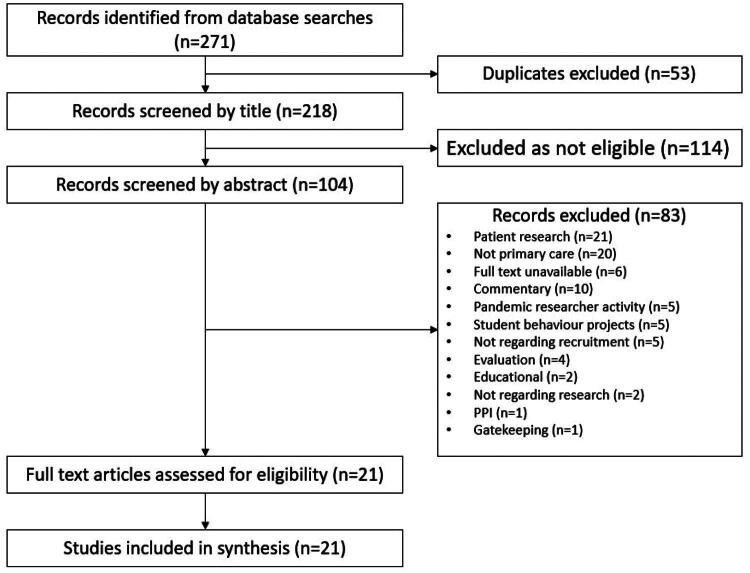
** ** A PRISMA diagram of study selection for barriers and facilitators to primary care staff taking part in research.

Database searches identified 271 records (see Figure 1). After duplicates were removed, titles were reviewed against the inclusion and exclusion criteria. Where there was a lack of clarity from the title, the abstract would be included. Abstracts of 104 papers were reviewed by ZE and 21 met the inclusion criteria. A Preferred Reporting Items for Systematic Reviews and Meta-Analyses (PRISMA) diagram is used to display the different stages of the review and reasons for non-inclusion of any studies (see Figure 1) [11]. The full text of 21 papers was reviewed by both ZE and MT and all were included.

### Characteristics of included studies

Characteristics of included studies can be found in Table 1. Studies were a mixture of qualitative interviews and focus groups and quantitative surveys with qualitative aspects. Participant numbers ranged between 11 and 1511. Studies originated all over the world with eight from the UK and five from the United States of America (USA), four elsewhere in Europe, two from Australia, one from Canada and one from Malaysia. Papers were published between 2000 and 2022. Seven studies were solely for GPs, one for pharmacists, one for administration staff, one for managers and the remainder were for a mixture of clinical and non-clinical roles. Nineteen papers explored barriers and facilitators and two solely looked at barriers. Five studies focused on retrospective barriers and/or facilitators to involvement in the studies they related to and the remaining 16 studies were regarding general barriers and/or facilitators to primary care staff conducting research.

**Table 1.** Characteristics and summary of the included studies.

Authors	Title	Year	Setting, Country	Methods	Participants	Barriers	Facilitators
Askew et al. [14]	General practice research: Attitudes and involvement of Queensland general practitioners.	2002	Primary care, Queensland, Australia	Qualitative and Quantitative. Postal questionnaire	467 GPs	Higher authority to clinical experience than research evidence. Doubts over credibility of researchers/research activity.	Academic mentors. Opportunities to participate in reputable, established and relevant research activities. Access to IT. Research useful for evidence-based medicine. Simple methodologies. Formal research training.
Bakken et al. [15]	Barriers, enablers, and incentives for research participation: a report from the Ambulatory Care Research Network (ACRN)	2009	Ambulatory care, Manhattan USA	Mixed methods – surveys, focus groups, interviews. To determine the level of interest in clinical research among community clinicians with barriers and facilitators	24 Surveys, 22 focus groups/ interviews. Ambulatory Care Network Physicians and Doctorally prepared nurse practitioners	Time. Lack of appropriate training. Inadequate compensation for time. Lack of backfill for clinical sessions (despite compensation). Lack of collaborators.	Relevance of research topic. Collaborators, mentors, research support staff. Potential to improve care. Opportunity for professional development. Patient and community engagement strategies.
Beckett et al. [16]	Bridging the gap between basic science and clinical practice: a role for community clinicians.	2011	Community healthcare, USA.	Qualitative. Review of literature and interviews with clinicians and stakeholders	Over 200 clinicians and other healthcare stakeholders from 2004-2005.	Pre-awareness - clinicians do not know about studies. Awareness - research questions not pertinent for their patients, too difficult to successfully implement in community practice. Information gathering - clinicians have insufficient information or ability to evaluate implications of participating. Maintenance - financial losses from research involvement, fear loss of patients to specialists, used for their patients.	Communication - multi-media campaign, better selling of studies, develop research principles. Explain study training, finances, protocols. Fair reimbursement, ethical principles to dissuade poaching. Encourage research community.
Befort et al. [17]	Perspectives on research among kansas county health department administrators	2009	Local health department administrators, Kansas, USA	Qualitative. Focus group	6 focus groups with 49 local health department administrators	Negative perception of research - too busy, a lot of work, complicated, boring, not reliable. Time, resources, relevance, skills. Lack of feedback. Lack of training. Lack of being ‘research minded’. Perception that researchers are out of touch as to what is needed. Language hard to understand. No ownership - feel researchers should ‘get their hands dirty’. Intimidating.	Seeing the value of information generated. Benefits include resources, opportunities for professional growth, networking, gaining information which informs practice/helps community. Money to increase staffing. Interesting to be part of improvements, exciting, new ideas and energy. More input and feedback.
Brandt et al. [18]	Federally Qualified Health Centres’ Capacity and Readiness for Research. Collaborations: Implications for Clinical-Academic-Community Partnerships	2015	Federally qualified health centres, USA	Online survey of experience, interest, partnerships, funding, barriers and facilitators.	14 representatives from health centres	Lack of dedicated time. Training to apply for and conduct research. Concern over loss of productivity. Methods to publish/disseminate Funding opportunities.	Improved patient outcomes and experience. Additional resources (including IT), Reduction in health disparities. Academic partnerships. Improved care delivery. Better access to specialty care.
Brodaty et al. [19]	Research in general practice: A survey of incentives and disincentives for research participation.	2013	Primary care, Sydney, Australia	Quantitative and qualitative survey. Questionnaire of barriers and incentives to GPs participating in research and brief interviews.	30 GPs − 10 involved in an Ageing in General Practice project – intervention and control arms and 10 who refused participation	Time. Paperwork. Inadequate explanation of research.	Desire to update knowledge. Altruism. Help patient. Payment. Importance of area of research.
Glynn et al. [20]	Research activity and capacity in primary healthcare: The REACH study: A survey.	2009, completed 2006	Primary care, Ireland	Quantitative and qualitative. Survey of research and development culture	Primary care 498 Health Care Professionals	Lack of protected time. Lack of funding. Lack of training, knowledge/research skills. Lack of supervision to support.	Positive attitude linked to previous research training. Currently involved in research. Not being a GP. Awareness of importance of research.
Gray et al. [21]	Barriers to the development of collaborative research in general practice: A qualitative study.	2001	Primary care, London UK	Qualitative interviews. Difficulties participating in research and suggestions for overcoming these	19 practices of differing research activity. 9 GPs, 4 practice managers, 3 practice nurses and 1 research assistant. 17 participants in total.	Lack of time. Varying demands of different studies (huge amount of work). Administrative staff get increased workload despite having least influence over decision to take part. Lack of ownership by doctors. Finance, IT, facilities issues. Partner vs employee. Lack of feedback from researchers. Unrealistic expectations of researchers. Flawed study protocols (lack of insight) - need more input from primary care in an earlier stage of study design.	Quantity of studies. Quality and timing of feedback about study outcomes makes difference between research being worthwhile and rewarding and feeling they have been used as a free source of patients, data, labour. Good communication (clear objectives and aware of primary care objectives and limitations).
Hange et al. [22]	Experiences of staff members participating in primary care research.	2015	Primary care, Sweden	Qualitative. Thematic analysis of focus group content.	5 focus groups of 4–8 nurses/GPs. 34 participants in total	Not a priority within organisation. No time to participate or communicate to other colleagues. Lack of communication with research team. Lack of support from research team. Little finance to cover. Lack of knowledge on the study area. Not knowing what happened.	Research is important. Only take part if line manager says to do so. Ownership of study if involved from outset.
Harrison [23]	Barriers and opportunities to developing research capacity in primary care trusts: The views of staff attached to a primary care trust.	2005	Primary care, Bolton, UK	Qualitative analysis of focus group.	46 PCT staff	Research not integral component of daily work. Managerial/organisational issues with developing capacity. Lack of appreciation and support by managers. Lack of structures to facilitate research.	Need to incorporate into routine work. Communication to ensure appropriate research done.
Hennrich et al. [24]	Effects of personalised invitation letters on research participation among general practitioners: A randomised trial.	2021	Primary care, Germany	Randomised trial of large observational study. Intervention group received personalised invitation to participate and control group a generic invite.	1511 GPs	Personalised invitation is not a barrier or facilitator.	
Hoffman et al. [25]	Clinician and Staff Perspectives on Participating in Practice-based Research (PBR): A Report from the Wisconsin Research and Education Network (WREN).	2015	Primary care, Wisconsin, USA	Qualitative focus groups at research network WREN. How has participation in practice-based research affected you and your clinic?	Focus groups in 2014 with 27 clinicians and clinic staff who participated in projects- physicians, nurses, managers, other clinical staff	Competing priorities. Time. System limitations. Not being fully informed of project expectations.	Project staff to do all. project-related procedures. Recognition of participation (publicly, CPD, certificates). Good relationship with study team.
Husin et al. [26]	Recruitment and participation of a survey in a public–private primary care setting: Experience from the QUALICOPC Malaysia.	2020	Primary care, Malaysia	Survey. Recruitment strategies, response rate and reasons for non-response for a study on quality and costs of primary care.	221 Public and 239 private primary care doctors	Too busy. Don’t feel have enough patients who would be interested. Didn’t find involvement beneficial.	
Jowett et al. [27]	Research in primary care: Extent of involvement and perceived determinants among practitioners from one English region.	2000	Primary care, West Midlands, UK	Quantitative and qualitative survey. Extent and determinants of GP research involvement.	1351 service GPs in West Midlands	Lack of time. Lack of staff to collect data. Lack of funding. Lack of interest. Lack of support. Lack of training.	Involvement in teaching. Research active partners. Protected time. Larger practice.
Lowrie et al. [28]	Research is ‘a step into the unknown’: An exploration of pharmacists’ perceptions of factors impacting on research participation in the NHS	2015	Primary and Secondary care, UK	Qualitative semi-structured interviews. NHS primary care and secondary care pharmacists’ perceptions and experiences of pharmacist-led research in the workplace.	54 pharmacists from general practices and secondary care in UK health authority	Lack of prioritisation. Lack of motivation, confidence, competence. Lack of practical support. Patient facing roles perceived as more important.	Part of training programme. Inherent value of research. Supportive line management.
Macfarlane et al. [29]	General practices as emergent research organisations: A qualitative study into organisational development	2005	Primary care, UK	Qualitative interviews. Historical accounts of the development of research activity.	28 key informants in 11 research practices	Conflicting priorities. Lack of time.	GP having interest in research and conducting adhoc, unfunded research. Research Champion (skill, interest, leadership, political clout). Grant. Academic training. Protected time. Networking. Joint academic/service appts. Accreditation.
MacLellan et al. [30]	Infrastructure challenges to doing health research ‘where populations with the most disease live’ in Covid times – A response to Rai et al.	2022	Primary care, UK	Qualitative interviews and quantitative survey. Interviews of healthcare staff and stakeholder, survey of patients. Paper describes barriers to research delivery and recruitment in CRN and mitigations.	11, Primary, urgent and emergency health centres and 111 sites	Infrastructure - CRN relationship with practice. CRN governance/efficiency.	GP champions. Stronger links with research active practice. Communication with practice teams to explain studies. Flexibility of interview formats. Weekly communication of recruitment figures to sites. Research nurse. Responsivity and proactive nature.
Salmon et al. [31]	Peering through the barriers in GPs’ explanations for declining to participate in research: The role of professional autonomy and the economy of time.	2007	Primary care, Liverpool, UK	Qualitative interviews with GPs who had declined to take part in a trial.	23 GPs	Lack of time. Perception that research was unethical (patients need GPs protection from researchers). Confidentiality, coercion. Not wanting to try new things. Lack of skills. Low status of research. Benefits would be elsewhere. Disinterest. Irrelevant to career/esteem. Not one of their responsibilities.	Money - could persuade GPs to use own time.
Stephenson et al. [32]	Barriers and facilitators to primary care research: Views of GP trainees and trainers.	2022	Primary care. NE and NW England	Online survey of barriers and facilitators to take part in research.	167 GP trainees and 140 trainers	Few trainers felt equipped to mentor trainees in research. Trainees had poor awareness of opportunities to take part. Trainees not aware of what research entailed.	Funded time for research. Role modelling. Trainees wanted to hear more about research opportunities.
Tawo et al. [33]	General practitioners’ willingness to participate in research: A survey in central Switzerland.	2019	Primary care, Lucerne, Switzerland	Postal survey and telephone contact with non-responders.	268 GPs	Expenses for time.	Relevance of research topic. Access to research network. Training.
Wozniak et al. [34]	A qualitative study examining healthcare managers and providers’ perspectives on participating in primary care implementation research.	2016	Primary care Alberta, Canada	Qualitative interviews. 34 face to face/telephone interviews with 17 managers.	17 PCN manager professionals, 34 interviews	Presumption that intervention better than usual care. Role conflict. Administrative burden. Perceptions of patient vulnerability. Perception that research was external to PCN. Lack of understanding of study design and ethics. Resources. Burden of having to recruit.	Perception that research was important.

IT: Information technology; PCT: Primary Care Trust; CPD: Continuing Professional Development; CRN: Clinical Research Network; PCN: Primary Care Network.

### Quality of included studies

The quality of included studies was assessed using the QuADS criteria by ZE and this was then reviewed by MT [12]. Results are displayed in Appendix 2.

Included studies were of varying quality although several of the lowest scoring papers were only short publications [21,27]. The highest scoring paper had rigorous, appropriate and clear methods and involved stakeholders throughout to strengthen its findings [29]. The lowest scoring paper was unclear in methods and participants although produced comprehensive modelling and potential solutions for barriers to primary care research [16]. Five of the papers had no mention of their aims and only seven were comprehensive in their explanation [16,18,21,30,34]. Only ten papers discussed involvement of stakeholders in their papers to strengthen their findings and three papers failed to mention any strengths and limitations of their work [14,18,21,25–31,34]. Although not a measure of the quality assessment tool, the age of the studies will influence the reliability of their findings due to the rapid changes in healthcare culture in recent times. The oldest study was from 2000 and the most recent was from 2022 [27,30,32].

Outcome measures in included studies were barriers to research, facilitators to research or both in some form. Some studies produced models of research culture or recommendations for future practice as a result of their findings [15–17,19,20,31,32].

### Thematic analysis of barriers and facilitators

Barriers and facilitators were identified from papers. Thematic analysis was used to identify five themes [13].

Facilitators were usually the inverse of the barriers, but these are discussed comprehensively below. Themes identified were from studies which included clinical staff, non-clinical staff or a combination. Clinical staff will be referred to as healthcare professionals, non-clinical staff as non-clinical staff and combination findings as staff. Where staff were in distinct groups, this is made clear.

Theme one is Research beliefs & understanding and is found in Box 1. Staff saw the benefits of research although perceived it to be an optional extra. They were more likely to get involved in interesting or relevant studies but were distrustful of researchers and their methods.

Theme two is Time, funding & recognition and can be found in Box 2. Time and how it would be funded were very important considerations in decisions as to whether to take part in research. Funding was often not adequate and backfill not available but studies were often unpredictable in resource need. Non-financial methods of recognition would also be beneficial.

Theme three is kills & Knowledge and can be found in Box 3. Previous research training gave staff the knowledge and skills to take part in research but these skills could easily be lost if not used regularly. Previous poor experience of research can prevent further involvement.

Theme four is Administration & Support and can be found in Box 4. There is a need for staff to have management support to take part in research. Administration support is essential along with ongoing support from the research team.

Theme five is Communication & People and is found in Box 5. Communication before the study can improve study design and provide realistic and flexible expectations of involvement. This communication should be maintained throughout involvement to prevent problems and language should be simple and accessible. Communication of study results are important to staff so they feel their contributions were worthwhile and valued. Affiliations with research active organisations were useful facilitators to research involvement.

Box 1 Theme one - Research beliefs & understanding

**1. Research beliefs & understanding**

The benefits of research are widely accepted even where staff were not able or did not want to participate [19,27,29,32,33]. Staff knew research had the potential to improve patient outcomes and were excited about being part of positive changes [25,27]. Although there was recognition that research led to improvements in healthcare, it was also seen as not belonging in primary care, not interesting or of low value [24,25,27,34]. Views about taking part in research activities due to altruism and because they were interested, despite the lack of funding, were also voiced [17,32]. Research is not commonly expected of staff working in primary care, so is seen as an optional extra to their role and a choice [27].

Staff were more likely to take part in research if it was relevant to their practice or they were interested in the subject area [18,23]. If they were interested, staff were sometimes unaware of where they could find opportunities which may be open to them [18,28]. Positive attitudes to research were linked to increased research activity [30].

There was a mistrust of researchers and doubts about credibility, ethics and misrepresentation of data by staff [19,23,25,29]. General misunderstanding of study design and a critique of methods by staff was voiced saying they were over-complicated and unnecessary [19,23,25]. Patient mistrust of research was also displayed and staff felt like they needed to protect patients from researchers [24,29]. This mistrust extended to the researchers themselves with a lack of understanding of the purpose of the research and the role of the researcher [15,25]. Some expressed a feeling that researchers did not fully understand the demands of general practice life, so did not design research that took this into account [15].

A lack of willingness to take part in research due to scepticism of new things was evident [24]. Some studies were thought to be unethical or irrelevant to general practice and people were more likely to take part in things which were seen as appropriate and useful for their patients [18,23,24,32]. Some staff felt general practice was being used for their patients and their data and lacked trust or understanding of its benefits [15,18].

There were some older studies which expressed more old-fashioned views, questioning how evidence-based practice could be compatible with patient-centred care and that involvement in research had the potential to harm their patients [24].

Box 2 Theme two – Time, funding & recognition

**2. Time, funding & recognition**

The majority of papers highlighted that a lack of time prevented participation in research [15–17,19,20,22,24,25,27,29–34]. Where staff had time which was protected on a regular basis, research could be prioritised rather than left at the bottom of the task list [17,20,27]. A commitment to research often meant less clinical time and the potential of getting behind with other duties [20]. Staff often felt conflicted as the long-term benefits of research are so great, yet the immediate pressures of general practice and the need to prioritise patients were keenly felt [17,19,22,27]. Clinicians in particular highlighted a perception that they had to choose between patient care and research, and that research was an additional burden on them [19–20]. The inability to work flexibly in primary care was a barrier, as this was often required when participating in research [27]. The erratic nature of general practice research activity does not allow for any sort of routine to be maintained and GPs believed that their days were already completely full and could not accommodate another activity, especially if it was not perceived as essential [33].

Part of the time theme is funding. Staff, including trainee staff, would be more likely to take part if there was funded time to do so [28]. Where funding was available to backfill clinical sessions, it was often difficult to utilise due to the pressures on the system and lack of spare clinical staff [29]. Staff who had previously had poor experiences of involvement in research studies which were time intensive and under-funded were less likely to participate in the future [18].

Sometimes research involved uncertain amounts of resources [18]. When agreeing to take part in a study it was not always clear what the time involvement was and whether any recompense was appropriate hence financial losses could result [18]. Financial incentives were more likely to make participation more attractive to GPs [32]. Financial gain seemed less important for the pharmacist population who may be less involved with the monetary sides of their roles [27]. Information technology (IT) and database access were discussed as well as limitations with electronic systems [15,22,23].

Non-financial recognition was also discussed. Some saw the potential for research to be a source of professional development or opportunity for professional growth [22,25,29]. Some people wanted more opportunities from research such as the ability to be involved in publications, dissemination or even joint clinical academic appointments [17,20]. Research in secondary care was seen as something which had the potential to further someone’s career whereas staff felt participation would not help a career in primary care as it was not something they would get any recognition for, whatever their role [24,27].

Box 3 Theme three - Skills & Knowledge

**3. Skills & Knowledge**

Lack of knowledge, skills or confidence in research involvement was a common theme [16,19,25,27–30,32]. Nurses felt they lacked skills and confidence with research, and that it was not part of their role hence were less likely to be involved [25,30]. Healthcare professionals who had experienced previous research training were more positive about being involved in research and this was seen as the most important driver for research [16,30]. Staff enrolled in postgraduate qualifications were often involved in research, but this did not always extend to maintaining involvement after the qualification was complete [27]. Healthcare professionals who were active members of grant applications were more likely to have had published work already and therefore more likely to have had some form of training [16,23]. The more experienced clinicians were, the more likely they were to take part in research due to training, qualifications or mentorship [27]. Lack of skills was mentioned in many studies by all staff – from trainees to those in more senior trainer roles [27,28]. There was a correlation between training and having an understanding of what being involved in primary care research meant, and where to find opportunities [28]. Research language was difficult to understand by some nurses and administrators and this was seen as a barrier [25,32]. Pharmacists also lacked research skills as these were not something picked up in their regular role and expressed concern that skills would be quickly lost if not frequently used [27]. Some clinicians cited bad experiences of research and lack of research integration into clinical practice [27,30]. Strong links with academic institutions were helpful to research activity as this would provide easy access to mentorship if needed and there was a desire for this from GPs [17,23]. Healthcare professionals were reluctant to take part in studies where they did not feel confident in the clinical area [33].

Box 4 Theme four - Administration & Support

**4. Administration & Support**

Support was discussed in many of the studies both in terms of administrative and structural support [15,16,27,33,34]. Research was not seen as a priority by managers which left teams feeling unmotivated and that the time spent doing research was not valued [27,34]. Research was not done if management had not sanctioned it, however important it was felt to be [33]. Lack of support staff was a barrier, although administrative staff felt they often had little influence over decisions to take part [15–16]. Administrative assistance to do time consuming data-related tasks was seen as integral to participation and had been helpful [22,29]. The burden of administration was felt keenly and tasks such as data entry, arranging appointments and research paperwork when staff are already overstretched was a barrier to taking part especially when staff had had previous bad experiences of research [16,19,32]. Research was seen as bureaucratic and unnecessarily complex and streamlining could help all involved [21].

Support from knowledgeable research-active colleagues within or external to the organisation (for example in a network) was something which was useful [27,32]. Where a research network was in place staff were sometimes unaware of its existence and, when accessed, support could be patchy and depend upon individual relationships [21,28]. Well-functioning research networks were helpful and had the potential to foster good working relationships and a feeling of belonging, as well as providing a source of research support [22]. Mentorship and peer support was something which could help guide people to make best use of their time and ensure staff did not feel isolated whilst undertaking research [23,29].

Support was also needed from research teams as well as experienced peers [30]. Once a study was agreed, research teams sometimes failed to give staff ongoing support leaving participants feeling overwhelmed and this could affect the recruitment, and therefore success of the study [33].

Box 5 Theme five - Communication & People

**5. Communication & People**

There was a correlation between communication and research activity engagement across a number of studies [15,18,25,34,35]. Before studies begin, communication about why the study was being carried out and relevance to patients and clinicians was lacking [25,32]. Conversations between researchers and clinicians about appropriateness of study design were often left until the last minute not leaving enough time to iron out potential problems before the study began [15,22]. Often this sort of collaboration just did not occur [19,25,33]. Clinicians felt insufficiently informed as to what participation in the study may mean for them and subsequently found researchers to have unrealistic expectations of what they could do [15,18,28]. Two-way communication about expectations, clear objectives and practice limitations led to successful collaboration and due to long studies and staff changes this may be needed repeatedly throughout the course of the study [15,33]. Language used in study documentation is also important, staff felt clear, accessible language could potentially lead to increased staff engagement and participation [23,25].

Post-study communication was seen as beneficial [25]. Staff liked to see how the data they worked hard to collect benefitted practice and what the study outcomes were [30]. This helped them feel that their participation was worthwhile, seen by some as a sort of reward and incentive to participate again [15].

Role modelling was seen as useful by trainees, but trainers sometimes felt ill-equipped to do this, which could stifle future involvement [28]. Having access to a research champion or research active partners may be beneficial and a good relationship with the study team was likely to lead to more success [16,17,29]. It was felt that there was a lack of ownership by GPs and other staff which disincentivised people to take part [15].

Both researchers and staff felt it was useful and necessary that they were able to adapt to circumstances they found themselves in [22]. The unpredictable nature of research and the inability to plan for it in advance meant that if researchers were flexible in their approach and staff were as flexible as their roles would allow, research was more likely to be successful [22].

## Discussion

This is the first review of barriers and facilitators to primary care staff conducting research and all studies found on this topic are included. The review found five different themes in relation to barriers and facilitators of primary care staff conducting research. Time, funding and research beliefs were common themes but training and support were also found to be facilitators. Good communication was a key facilitator at all points of the research journey.

Beliefs about research were important and although staff knew that research was a positive thing for patients, they still held some beliefs that research was not something that needed to be done. All papers talked about time being a barrier but it could also be a facilitator if protected time could be provided. A multi-national study looking at physician’s lack of control over their time found that physicians in particular felt they had the least amount of control over their time [35]. This then translates to being a barrier for research as it is something that is seen as ‘extra’ to the rest of the role and not an immediate necessity in primary care. This finding echos what was found in this study where time was cited as a barrier in the majority of the papers.

Training and knowledge about research in general, ethics, research procedures and ongoing training on individual studies would be beneficial to improve confidence in carrying out research studies. Funding and recognition for doing research was important as often research is carried out at a loss to the business making it less attractive. Many research activities require administrative time so support from administrative staff makes research much more achievable as well as support from experienced peers or research active groups. As research in primary care is not yet embedded, it is also perhaps not surprising that research support and administration are not yet roles which are commonplace. The emergence of this role was documented as long ago as 1959 and its development throughout the late 90’s and early 2000s was more in an academic context [36–37]. The results of this paper highlight the need for research administration roles to facilitate research conduct in the primary care setting.

Communication at all points of the research journey helps to address concerns and make staff feel confident and that their involvement was worthwhile. This is not something that is at the forefront of any of the papers analysed or a theme from the wider literature. Clinical and non-clinical staff need better communication before, during and after studies. The latter would help staff feel like their contribution was valued and this may affect their willingness to take part in future studies. The importance of dissemination is widely known but the need for effective communication with those conducting the research throughout the study period is novel.

An adaptive and supportive team were useful in research involvement as studies were often unpredictable in nature. To the author’s knowledge, no frameworks currently exist on primary care healthcare staff research involvement in research.

### Implications for practice

Primary care management should place more importance on research and include it as part of staff’s personal development plans to encourage participation [23,25]. The inclusion of non-GP research expertise in primary care could potentially improve research capacity [32]. Research activity should be shared in partnerships but inclusion in general practice contracts and development of a framework would help to embed it into everyday practice [23]. Beckett developed a model of how clinicians decide to take part in research starting with pre-awareness of opportunities, followed by awareness and information gathering where they are deciding the implications of taking part then first protocol where they have their first experiences of participating and maintenance [16]. Clinicians can develop concerns at all stages, for example they may have a poor first experience which may put them off any future participation. Solutions at each stage can be addressed through improved communication. This enhanced communication throughout the research process was recommended by several authors [14,15,19,21,22,25,30]. Multiple studies recommended the inclusion of primary care staff in the development of research protocols to ensure activities were efficient, achievable and appropriate [14,19,22,25,30,34]. Continued clear and concise two-way communication after staff had agreed to take part was recommended to maintain momentum, ensure staff felt supported and encourage successful completion [21–22].

Training of staff was recommended both before studies commenced and on an ongoing basis to address knowledge gaps and low confidence amongst non-clinical and clinical staff [17–18]. This training would improve efficiency of studies and reduce their burden on staff and practice activity. Improvements in funding for study participation were recommended to give appropriate recompense for time taken to complete research work [19,21].

### Strengths and limitations

This review has a number of strengths. To our knowledge, this is the first systematic review of its kind to explore the experiences of primary care staff surrounding the barriers and facilitators of taking part in research activities. The review was structured, with a clear research question, methods and search strategy to allow for trustworthiness and usefulness of results [38]. The quality of the included studies was variable and some included studies were not full papers which could affect the reliability of findings. Qualitative data extraction can be prone to bias however trustworthiness and rigour may be more important in systematic reviews of this nature [39]. The latter are demonstrated by the robust processes undertaken during data extraction and interpretation.

## Conclusion

Multiple barriers and facilitators exist to primary care staff conducting research. Good communication at all stages would go a long way to enabling a research-active culture. This should start at the research design stage by involving primary care staff in design and ensuring processes are aligned to current practices. Opportunities to be involved in research should be shared, including regular communication with primary care staff. Ensuring that researchers are clear about how involvement can have a positive impact on patients or general practice more widely can increase the likelihood of staff engagement and participation. Continued two-way communication during the study can help to iron out any problems before they start to affect the success of the research and after completion can help staff to understand what they have contributed, how it affects the overall study and also helps them to feel like their hard work was worth it.

Basic, simple research training for all staff at all levels would ensure understanding of processes, ethics and improve confidence. Research should be embedded into the whole primary care team so responsibility lies with the most appropriate people and those people should have protected time to carry it out. Successful research in primary care allows for positive progress and improved care for patients.

## Supplementary Material

Supplemental Material

## References

[1] NHS. The NHS long term plan. London: NHS; 2019. [accessed 2023 November 11] https://www.longtermplan.nhs.uk/online-version/

[2] NHS England. Primary care networks. 2023. [accessed 2023 October 16] www.england.nhs.uk/primary-care/primary-care-networks/

[3] Jonker L, Fisher SJ. The correlation between National health service trusts’ clinical trial activity and both mortality rates and care quality commission ratings: a retrospective cross-sectional study. J Public Health. 2018;157:1–6. doi: 10.1016/j.puhe.2017.12.022. 29438805

[4] Jonker L, Fisher SJ, Dagnan D. Patients admitted to more research-active hospitals have more confidence in staff and are better informed about their condition and medication: results from a retrospective cross-sectional study. J Eval Clin Pract. 2020;26(1):203–208. doi: 10.1111/jep.13118. 30784152

[5] Harding K, Lynch L, Porter J, et al. Organisational benefits of a strong research culture in a health service: a systematic review. Aust Health Rev. 2016;41(1):45–53. doi: 10.1071/AH15180. 27074113

[6] Manchester University NHS Foundations Trust. Research. 2023. [accessed 20203 November 11] https://mft.nhs.uk/research/

[7] Askew DA, Glasziou PP, Del Mar CB. Research output of Australian general practice: a comparison with medicine, surgery and public health. Med J Aust. 2001;175(2):77–80. doi: 10.5694/j.1326-5377.2001.tb143534.x. 11556423

[8] NHS Confederation. 2022. Primary care networks: three years. [accessed 20203 October 16] www.nhsconfed.org/publications/pcns-three-years

[9] NIHR. 2023b. Under-served communities. [accessed 2023 October 16]. https://www.nihr.ac.uk/about-us/our-key-priorities/under-served-communities.htm

[10] Grimethorpe Surgery. 2022. Most GPs exceed safe limit for appointments every working day. [accessed 2023 November 11] https://www.grimethorpesurgery.nhs.uk/2022/12/23/most-gps-exceed-safe-limit-for-appointments-every-working-day-poll-reveals/

[11] Liberati A, Altman DG, Tetzlaff J, et al. The PRISMA statement for reporting systematic reviews and meta-analyses of studies that evaluate healthcare interventions: explanation and elaboration. BMJ. 2009;339:b2700. doi: 10.1136/bmj.b2700. 19622552 PMC2714672

[12] Harrison R, Jones B, Gardner P, et al. Quality assessment with diverse studies (QuADS): an appraisal tool for methodological and reporting quality in systematic reviews of mixed- or multi-method studies. BMC Health Serv Res. 2021;21:144. doi: 10.1186/s12913-021-06122-y. 33588842 PMC7885606

[13] Braun V, Clarke V. Using thematic analysis in psychology. Qual Res Psychol. 2006;3(2):77–101. doi: 10.1191/1478088706qp063oa.

[14] Askew DA, Clavarino AM, Glasziou PP, et al. General practice research: attitudes and in. Involvement of Queensland general practitioners. Med J Aust. 2002;177(2):74–77. doi: 10.5694/j.1326-5377.2002.tb04670.x. 12098342

[15] Bakken S, Lantigua RA, Busacca LV, et al. Barriers, enablers, and incentives for research participation: a report from the Ambulatory Care Research Network (ACRN). J Am Board Fam Med. 2009;22(4):436–445. doi: 10.3122/jabfm.2009.04.090017. 19587259 PMC2744643

[16] Beckett M, Quiter E, Ryan G, et al. Bridging the gap between basic science and clinical practice: a role for community clinicians. Implement Sci. 2011;6(35):1–10. 10.1186/1748-5908-6-34 PMC3087703 21463516

[17] Befort CA, Or S, Davis A, et al. Perspectives on research among Kansas county health department administrators. J Public Health Manag Pract. 2009;15(3):9–15. 10.1097/01.PHH.0000349745.49856.2e PMC3954814 19363394

[18] Brandt HM, Young VM, Campbell DA, et al. Federally Qualified Health Centers’ Capacity and Readiness for Research. Collaborations: implications for Clinical-Academic-Community Partnerships. Clin Transl Sci. 2015;8(4):391–393. doi: 10.1111/cts.12272. 25962873 PMC4553115

[19] Brodaty H, Gibson LHR, Waine ML, et al. Research in general practice: a survey of incentives and disincentives for research participation. Ment. Health Fam Med. 2013;10:163–173. 24427184 PMC3822664

[20] Glynn LG, O’Riordan C, MacFarlane A, et al. Research activity and capacity in primary healthcare: the REACH study: a survey. BMC Fam Pract. 2009;10(33):1–7. 10.1186/1471-2296-10-33 PMC2684072 19432990

[21] Gray RW, Woodward NJ, Carter YH. Barriers to the development of collaborative research in general practice: a qualitative study. Br J Gen Pract. 2001;51:221–222. 11255904 PMC1313954

[22] Hange D, Bjorkelund C, Svenningsson I, et al. Experiences of staff members participating in primary care research. Int J Gen Med. 2015;8:143–148. doi: 10.2147/IJGM.S78847. 25926753 PMC4403682

[23] Harrison RA. Barriers and opportunities to developing research capacity in primary care trusts: the views of staff attached to a primary care trust. Prim Health Care Res Dev. 2005;6(3):185–189. doi: 10.1191/1463423605pc233oa.

[24] Hennrich P, Arnold C, Wensing M. Effects of personalised invitation letters on research participation among general practitioners: a randomised trial. BMC Med Res Meth. 2021;21:247. 10.1186/s12874-021-01447-y PMC8590365 34773971

[25] Hoffman AE, Leege EK, Plane MB, et al. Clinician and Staff Perspectives on Participating in Practice-based Research (PBR): a Report from the Wisconsin Research and Education Network (WREN). J Am Board Fam Med. 2015;28:639–648. 26355136 10.3122/jabfm.2015.05.150038 PMC4934613

[26] Husin M, Rahman N, Wong XC, et al. Recruitment and participation of a survey in a public-private primary care setting: experience from the QUALICOPC Malaysia. Prim. Health Care Res Dev. 2020;21(e51):1–8. 10.1017/S1463423620000511 PMC7681175 33213564

[27] Jowett SM, Macleod J, Wilson S, et al. Research in primary care: extent of involvement and perceived determinants among practitioners from one English region. Br J Gen Pract. 2000;50:387–389. 10897537 PMC1313704

[28] Lowrie R, Morrison G, Lees R, et al. Research is ‘a step into the unknown’: an exploration of pharmacists’ perceptions of factors impacting on research participation in the NHS. BMJ Open. 2015;5(12):e009180. doi: 10.1136/bmjopen-2015-009180. PMC4710811 26719315

[29] Macfarlane F, Shaw S, Greenhalgh T, et al. General practices as emergent research organizations: a qualitative study into organizational development. Fam Pract. 2005;22(3):298–304. doi: 10.1093/fampra/cmi011. 15805134

[30] MacLellan J, Turnbull J, Pope C. Infrastructure challenges to doing health research “where populations with the most disease live” in Covid times-a response to Rai et al. BMC Med Res Meth. 2022;22:265. 10.1186/s12874-022-01737-z PMC9547085 36209066

[31] Salmon P, Peters S, Rogers A, et al. Peering through the barriers in GPs’ explanations for declining to participate in research: the role of professional autonomy and the economy of time. Fam Pract. 2007;24(3):269–275. doi: 10.1093/fampra/cmm015. 17504773

[32] Stephenson S, Tang EYH, Tang E, et al. Barriers and facilitators to primary care research: views of GP trainees and trainers. Brit J Gen Pract. 2022;6(2):30BJGPO.2021.0099. doi: 10.3399/BJGPO.2021.0099. PMC9447324 35135815

[33] Tawo S, Gasser S, Gemperli A, et al. General practitioners’ willingness to participate in research: a survey in central Switzerland. PLoS One. 2019;14(3):e0213358. doi: 10.1371/journal.pone.0213358. 30822332 PMC6396922

[34] Wozniak LA, Soprovich A, Rees S, et al. A qualitative study examining healthcare managers and providers’ perspectives on participating in primary care implementation research. BMC Health Serv Res. 2016;14:316. 10.1186/s12913-016-1577-1 PMC4965883 27473755

[35] Konrad T, Link CL, Shakleton RJ, et al. It’s about time: physicians’ perceptions of time constraints in primary care medical practice in three national healthcare systems. Medical Care. 2010;48(2):95–100. doi: 10.1097/MLR.0b013e3181c12e6a. 20057331 PMC3621071

[36] Kaplan N. The role of the research administrator. Admin Sci Quar. 1959;4(1):20–42. doi: 10.2307/2390647.

[37] Kerridge S, Scott SF. Research Administration around the World. Res Manag Rev. 2018;23(1):1–34.

[38] Butler A, Hall H, Copnell B. A guide to writing a qualitative systematic review protocol to enhance evidence-based practice in nursing and health care. Worldv Evid Based Nurs. 2016;13(3):241–249. doi: 10.1111/wvn.12134. 26790142

[39] Galdas P. Revisiting bias in qualitative research: reflections on its relationship with funding and impact. Int J Qual Meth. 2017;16(1):1–2. doi: 10.1177/1609406917748992.

