# Correction

**DOI:** 10.1080/13814788.2026.2633044

**Published:** 2026-02-23

**Barriers and facilitators to primary care staff conducting research – a qualitative systematic review**

Z. Edwards and M. Tatterton

*European Journal of General Practice,* Volume 31, Issue 1, 2539777

DOI: 10.1080/13814788.2025.2539777

When the above article was first published an error in reference numbering led to reference [24] (Hennrich P, Arnold C, Wensing M. Effects of personalised invitation letters on research participation among general practitioners: a randomised trial. BMCMed Res Meth. 2021;21:247) being incorrectly cited in the text in the place of reference [31] (Salmon P, Peters S, Rogers A, et al. Peering through the barriers in GPs’ explanations for declining to participate in research: the role of professional autonomy and the economy of time. Fam Pract. 2007;24(3):269–275. doi: 10.1093/fampra/cmm015).

The error was introduced during revisions following the peer review process and has now been corrected in the online version.

