# Supplementary material for: Barriers and facilitators to primary care staff conducting research – a qualitative systematic review
Source: Eur J Gen Pract. 2025 Aug 13;31(1):2539777. doi: 10.1080/13814788.2025.2539777 (PMC12351701; doi:10.1080/13814788.2025.2539777)
Supplement: Supplemental Material [file IGEN_A_2539777_SM0542.zip › ejgp-2024-0243-20250723192534/suppl_data/ejgp-2024-0243-File004.docx]

**Appendix 1**

**Search strategy employed in systematic review**

| Search number | Search terms |
| --- | --- |
| #1 | challenges OR barriers OR difficulties OR problems OR limitations |
| #2 | facilitators OR motivators OR enablers |
| #3 | #1 OR #2 |
| #4 | gp OR ‘general practice’ OR ‘general practitioner’ OR primary medical care’ OR primary AND care OR family AND doctor |
| #5 | primary care OR ‘primary health care’ OR primary health care OR ‘general practice’ |
| #6 | ‘pharmacist’ OR ‘nurse’ OR allied AND health AND profession OR ‘paramedical profession’ |
| #7 | ‘administrative personnel’ OR ‘management’ OR ‘office worker’ OR ‘staff’ |
| #8 | clinician OR health care personnel OR nurse OR physician OR doctor |
| #9 | #4 OR #5 OR #6 OR #7 OR #8 |
| #10 | #3 AND #9 |
| #11 | ‘research’ OR ‘study’ |
| #12 | #10 AND #11 |
| #13 | #4 OR #6 OR #7 OR #8 |
| #14 | #3 AND #13 AND #11 AND #5 |
| #15 | ‘research participation’ OR ‘research activities’ |
| #16 | #3 AND #13 AND #5 AND #15 |
