# Supplementary material for: Barriers and facilitators to primary care staff conducting research – a qualitative systematic review
Source: Eur J Gen Pract. 2025 Aug 13;31(1):2539777. doi: 10.1080/13814788.2025.2539777 (PMC12351701; doi:10.1080/13814788.2025.2539777)
Supplement: Supplemental Material [file IGEN_A_2539777_SM0542.zip › ejgp-2024-0243-20250723192534/suppl_data/ejgp-2024-0243-File005.docx]

**Appendix 2 A table showing the quality of included papers using the QuADS criteria [12]**

|  | **1.Theoretical underpinning** | **2.Research aims** | **3.Setting & population** | **4.Appropriate design** | **5.Appropriate sampling** | **6.Choice data collection** | **7.Content data collection** | **8.Descriiption procedure** | **9.Recruitment data** | **10. Analysis justification** | **11.Method of analysis** | **12.Research stakeholders** | **13.Strengths/limitations** | **TOTAL** |
| --- | --- | --- | --- | --- | --- | --- | --- | --- | --- | --- | --- | --- | --- | --- |
| **Askew et al (2002)[14]** | 1 | 2 | 2 | 2 | 2 | 1 | 2 | 2 | 2 | 1 | 3 | 1 | 2 | **23** |
| **Bakken et al (2009)[15]** | 1 | 1 | 3 | 3 | 2 | 3 | 3 | 3 | 3 | 1 | 2 | 0 | 2 | **27** |
| **Beckett et al (2011)[16]** | 0 | 0 | 1 | 0 | 1 | 0 | 1 | 1 | 0 | 1 | 1 | 0 | 1 | **7** |
| **Befort et al (2009)[17]** | 2 | 2 | 3 | 3 | 2 | 2 | 3 | 2 | 1 | 1 | 3 | 2 | 2 | **28** |
| **Brandt et al (2015)[18]** | 1 | 0 | 1 | 0 | 1 | 2 | 1 | 1 | 1 | 0 | 2 | 2 | 0 | **12** |
| **Brodarty et al (2013)[19]** | 2 | 3 | 3 | 3 | 3 | 3 | 3 | 1 | 2 | 2 | 2 | 0 | 1 | **28** |
| **Glynn et al (2009)[20]** | 1 | 3 | 3 | 2 | 2 | 2 | 2 | 3 | 3 | 3 | 2 | 0 | 3 | **29** |
| **Gray et al (2001)[21] Short report** | 1 | 0 | 3 | 0 | 1 | 2 | 0 | 1 | 0 | 0 | 2 | 0 | 0 | **10** |
| **Hange et al (2015)[22]** | 2 | 3 | 2 | 2 | 2 | 1 | 2 | 2 | 1 | 3 | 2 | 0 | 2 | **24** |
| **Harrison (2005)[23]** | 1 | 1 | 1 | 1 | 1 | 1 | 2 | 2 | 1 | 0 | 2 | 0 | 1 | **14** |
| **Hennrich et al (2021)[24]** | 3 | 3 | 2 | 3 | 1 | 2 | 2 | 3 | 3 | 2 | 3 | 0 | 2 | **29** |
| **Hoffman et al (2015)[25]** | 2 | 1 | 3 | 2 | 2 | 0 | 2 | 3 | 2 | 2 | 2 | 2 | 3 | **26** |
| **Husin et al (2020)[26]** | 2 | 3 | 3 | 1 | 3 | 2 | 1 | 1 | 2 | 2 | 2 | 3 | 1 | **26** |
| **Jowett et al (2000)[27] Brief report** | 0 | 1 | 2 | 1 | 1 | 0 | 1 | 1 | 2 | 2 | 2 | 0 | 0 | **13** |
| **Lowrie et al (2015)[28]** | 3 | 1 | 2 | 3 | 2 | 3 | 3 | 2 | 2 | 2 | 2 | 1 | 2 | **28** |
| **Macfarlane et al (2005)[29]** | 1 | 3 | 3 | 3 | 3 | 3 | 3 | 3 | 2 | 3 | 3 | 3 | 1 | **34** |
| **MacLellan et al (2022)[30]** | 1 | 0 | 3 | 0 | 1 | 0 | 0 | 0 | 0 | 0 | 0 | 3 | 3 | **11** |
| **Salmon et al (2007)[31]** | 2 | 2 | 3 | 2 | 1 | 2 | 2 | 2 | 1 | 1 | 3 | 1 | 3 | **25** |
| **Stephenson et al (2022)[32]** | 1 | 3 | 3 | 2 | 2 | 0 | 1 | 2 | 2 | 2 | 2 | 0 | 2 | **22** |
| **Tawo et al (2018)[33]** | 2 | 2 | 2 | 1 | 1 | 2 | 1 | 2 | 1 | 1 | 2 | 0 | 3 | **20** |
| **Wozniak et al (2016)[34]** | 2 | 0 | 3 | 0 | 3 | 1 | 2 | 3 | 0 | 2 | 3 | 3 | 1 | **23** |
