# Supplementary material for: Barriers and facilitators to primary care staff conducting research – a qualitative systematic review
Source: Eur J Gen Pract. 2025 Aug 13;31(1):2539777. doi: 10.1080/13814788.2025.2539777 (PMC12351701; doi:10.1080/13814788.2025.2539777)
Supplement: Supplemental Material [file IGEN_A_2539777_SM0542.zip › ejgp-2024-0243-20250723192534/graphic/ejgp-2024-0243-File002.docx]

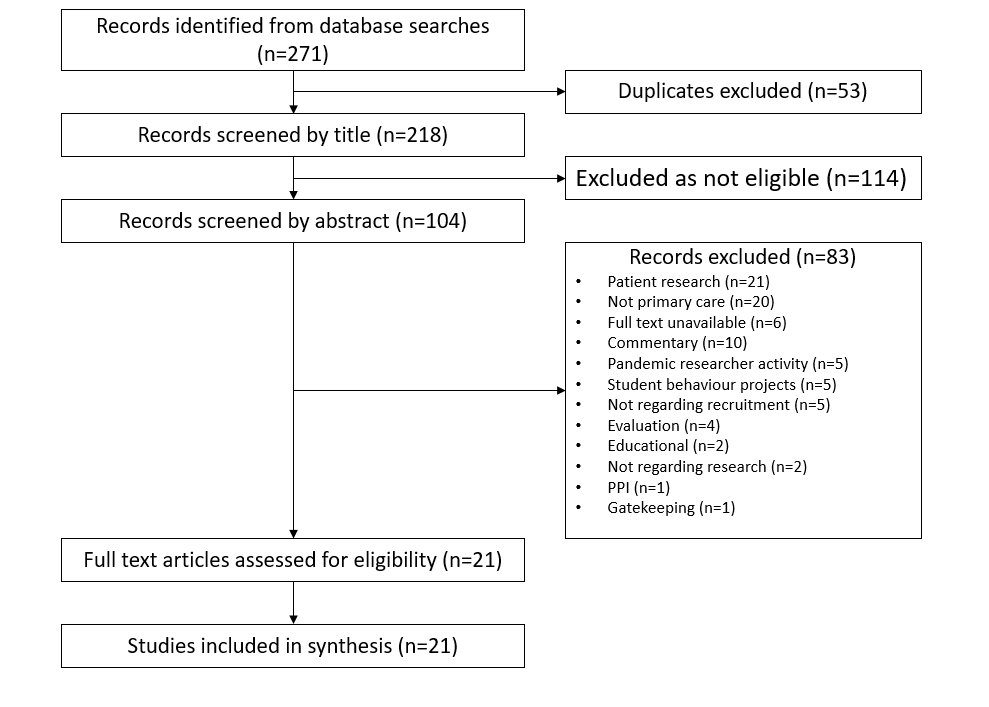


**Figure 1 A PRISMA diagram of study selection for barriers and facilitators to primary care staff taking part in research**
