# Supplementary material for: Barriers and facilitators to primary care staff conducting research – a qualitative systematic review
Source: Eur J Gen Pract. 2025 Aug 13;31(1):2539777. doi: 10.1080/13814788.2025.2539777 (PMC12351701; doi:10.1080/13814788.2025.2539777)
Supplement: Supplemental Material [file IGEN_A_2539777_SM0542.zip › ejgp-2024-0243-20250723192534/ejgp-2024-0243-manifest.html]

Manifest file for export ejgp-2024-0243-20250723192534


|  |  |
| --- | --- |
| File name: ejgp-2024-0243-20250723192534 | |
| Export Date: 23-Jul-2025 | |
| Output Format: XML (ScholarOne DTD) | |
| ejgp-2024-0243-20250723192534/doc/ejgp-2024-0243-File001.docx | Version 1.0 |
| ejgp-2024-0243-20250723192534/graphic/ejgp-2024-0243-File002.docx | Version 1.0 |
| ejgp-2024-0243-20250723192534/suppl\_data/ejgp-2024-0243-File003.docx | Version 1.0 |
| ejgp-2024-0243-20250723192534/suppl\_data/ejgp-2024-0243-File004.docx | Version 1.0 |
| ejgp-2024-0243-20250723192534/suppl\_data/ejgp-2024-0243-File005.docx | Version 1.0 |
| ejgp-2024-0243-20250723192534/pdf/ejgp-2024-0243.pdf |  |
| ejgp-2024-0243-20250723192534/ejgp-2024-0243-metadata.xml |  |
| ejgp-2024-0243-20250723192534/s1.dtd |  |
| manifest.html | This document |
